# Vigorous Root Growth Is a Better Indicator of Early Nutrient Uptake than Root Hair Traits in Spring Wheat Grown under Low Fertility

**DOI:** 10.3389/fpls.2016.00865

**Published:** 2016-06-16

**Authors:** Yaosheng Wang, Kristian Thorup-Kristensen, Lars Stoumann Jensen, Jakob Magid

**Affiliations:** ^1^Institute of Environment and Sustainable Development in Agriculture, Chinese Academy of Agricultural SciencesBeijing, China; ^2^Plant and Soil Science Section, Department of Plant and Environmental Sciences, University of CopenhagenFrederiksberg, Denmark; ^3^Crop Science Section, Department of Plant and Environmental Sciences, University of CopenhagenTaastrup, Denmark

**Keywords:** early growth stage, wheat genotype, low nutrient availability, nutrient uptake, root system

## Abstract

A number of root and root hair traits have been proposed as important for nutrient acquisition. However, there is still a need for knowledge on which traits are most important in determining macro- and micronutrient uptake at low soil fertility. This study investigated the variations in root growth vigor and root hair length (RHL) and density (RHD) among spring wheat genotypes and their relationship to nutrient concentrations and uptake during early growth. Six spring wheat genotypes were grown in a soil with low nutrient availability. The root and root hair traits as well as the concentration and content of macro- and micronutrients were identified. A significant genetic variability in root and root hair traits as well as nutrient uptake was found. Fast and early root proliferation and long and dense root hairs enhanced uptake of macro- and micronutrients under low soil nutrient availability. Vigorous root growth, however, was a better indicator of early nutrient acquisition than RHL and RHD. Vigorous root growth and long and dense root hairs ensured efficient acquisition of macro- and micronutrients during early growth and a high root length to shoot dry matter ratio favored high macronutrient concentrations in the shoots, which is assumed to be important for later plant development.

## Introduction

Nutrient availability is the primary limitation to nutrient uptake and crop productivity in low-input and organic agriculture (Mueller et al., 2012). It has been suggested that the development of genotypes with appropriate root traits might increase crop yields on infertile soils (Lynch, 2007, 2011; Hawkesford, 2011), and modern cultivars that were bred for the conventional high-input sector with selection in conventional breeding programmes lack important traits required under low-input and organic production conditions (Van Bueren et al., 2002; Murphy et al., 2007; Wolfe et al., 2008). According to Parr et al. (1990), low input farming systems “seek to optimize the management and use of internal production inputs, and minimize the use of external production inputs.”, and organic farming generally adheres to this principle. In the Danish organic farming sector it is currently a challenge to ensure a high quality of bread wheat for baking due to a low nutrient content in the grain. Further, limitations may yet be imposed on nutrient import in organic farming systems, as exemplified by the plan in Denmark to phase out the use of manures and crop residues from conventional farms (Oelofse et al., 2013), and may be expected to exacerbate the challenge for adequate plant nutrition. Therefore, there is an interest in elucidating the variability between genotypes of spring wheat in their efficiency in utilizing soil with lower fertility.

Root proliferation is critical for plants in exploiting the soil volume for uptake of mineral elements, and root length has been shown to be especially important for less mobile nutrients (Barber, 1995; Lynch, 2013; White et al., 2013). During early growth stages, root density in upper soil layers develops fast, and reaches so high root density there that the root to root competition becomes high, and there may be little gain by further increase in root density. However, until this stage is reached, the root density is low, and exploitation of water and nutrients from even the upper soil layers is still limited by low root density. During this early growth stage increased root density can therefore be important for vigorous shoot growth and establishment of the crop and its yield potential, especially on soils with low nutrient content. Early nutrient acquisition may be a challenge in stockless organic farming systems, especially in cool temperate zones (Oelofse et al., 2013).

Large differences in root morphology have been observed between crop species genotypes (O'Toole and Bland, 1987; Romer et al., 1988; Bruck et al., 2003). Wheat and barley genotypes with increased root hair length (RHL) and density (RHD) have been associated with greater capacity for uptake of soil phosphorus (P; Gardiner and Christensen, 1990; Gahoonia and Nielsen, 1996, 1997; Gahoonia et al., 1999; Brown et al., 2012) and higher yield potential (Gahoonia and Nielsen, 2004) under P-limited condition. Much of the research on nutrient acquisition and root hairs has been related to P uptake, but root hair development has also been shown to be regulated by a much more mobile nutrient like nitrate (Bhat et al., 1979; Foehse and Jungk, 1983), and other more immobile nutrients like iron (Schmidt et al., 2000), manganese and zinc (Ma et al., 2001).

While root hair traits have been considered important for uptake of especially less mobile nutrients as discussed above, deeper rooting associated with vigorous root growth has been shown to be important for uptake of water and nitrogen especially in the nitrate form (Liao et al., 2006; Palta et al., 2007; Palta and Watt, 2009; Lynch, 2013). In a recent paper White et al. (2013) discussed root ideotypes for improving the acquisition of essential mineral elements, and identified root growth vigor as well as RHL and RHD as globally important traits affecting the uptake of most essential nutrients. Liao et al. (2006) and Palta et al. (2007) have shown that in some cases there is a positive relationship between vigorous shoot and root growth, so it would be possible to imagine an ideotype with a vigorous initial growth of roots and shoots, coupled with an efficient uptake of limited soil nutrients. Thus, it is relevant to assess the variability of root traits and the associated uptake of nutrients in existing cultivars to be used for low input production system.

Until recently root growth and function have rarely been used as selection criteria in breeding programmes (Lynch and Brown, 2012). Measuring root parameters is laborious, and optimal root function is a highly complex topic, so it has been difficult to define precisely what to measure and select for. Since early crop development is crucial in development of yield potential, the aims of the present study were to: (1) investigate the genetic variations in root and root hair traits among spring wheat genotypes during early growth stages in a low fertility soil, and (2) to examine their importance for uptake of macro- and micronutrients.

## Materials and methods

### Selection of genotypes

A preliminary experiment was conducted to screen root trait responses of 19 spring wheat (*Triticum aestivum* L.) genotypes representing both recent and older genotypes to low P concentration under hydroponic growth conditions (Table S1). The nutrient solution was composed of 2 μM KH_2_PO_4_, 0.2 mM K_2_SO_4_, 0.3 mM MgSO_4_·7H_2_O, 0.1 mM NaCl, 0.3 mM Mg (NO_3_)_2_·6H_2_O, 0.9 mM Ca (NO_3_)_2_·4H_2_O, 0.6 mM KNO_3_, 1 μM MnCl_2_·4H_2_O, 0.8 μM Na_2_MoO_4_, 0.7 μM ZnCl_2_, 0.8 μM CuSO_4_·5H_2_O, 2 μM H_2_BO_3_, 1 μM NiSO_4_·6H_2_O, and 50 μM Fe-EDTA. The culture solutions were continually aerated and their pH was maintained between 5.8 and 6.0 with daily additions of KOH and HCl. Based on their root length and RHL after 24 days growth at P limiting (2 μM P) condition (Table S2) we selected six genotypes (Table 1) with weaker or stronger expression of these traits. The seed dry matter (DM) for A35-213, Farah, April Bearded, Hindy62, Hankkijan Tapio and Dacke was 31, 42, 34, 47, 43, and 34 mg, respectively.

**Table 1:** **Identities of spring wheat genotypes used in the experiment**.

**Genotype**	**NordGen accession number**	**Origin**	**Released year**
A35–213	NGB2144	Norway	Unknown
Farah	NGB4837	Afghanistan	Unknown
April Bearded	NGB8212	United Kingdom	1838
Hindy62	NGB8921	Egypt	Unknown
Hankkijan Tapio	NGB13344	Finland	1980
Dacke	NGB9955	Sweden	1990

### Plant growth trials

The spring wheat varieties were grown in soil filled Perspex tubes under a glass roof shelter at the experimental farm of Department of Plant and Environmental Sciences, University of Copenhagen, Taastrup. The average, maximum, and minimum temperature were 16, 19, and 11°C, respectively, during the experimental period. The tubes were 0.5-m high PVC tubes with an inner diameter of 14.5 cm. Tube bottoms were sealed with mesh, allowing free drainage. There was no water drainage during the experimental period. The tubes were filled with 11.7 kg of air-dried soil-sand mixture to arrive at a bulk density of 1.4 g cm^−3^. Prior to the plant growth trials, it was established that the mixture of soil and purified sand allowed isolation of roots and adhering root hairs with minimal damage to the root system. Examples of root hair images analyzed for root hair measurement were shown in Figure S1.

Soil for the experiment was collected from the upper horizon (0–30 cm) in a long-term field trial, from a treatment which has not been fertilized but grown with cereals including undersown clover grass mixture for green manure since 2003 at the experimental farm of University of Copenhagen (Poulsen et al., 2013). In the years before sampling the yields of cereal crops grown in this plot were low (50–55%) compared to the corresponding plot fertilized with 90 kg N in NPK (21-3-10). It was classified as sandy loam with total C of 14.8 g kg^−1^, total N of 1.52 g kg^−1^, total P of 0.28 g kg^−1^, Olsen P of 18 mg kg^−1^, and total K of 17.6 g kg^−1^. The soil taken was air-dried, passed through a 2-mm sieve and thoroughly mixed with an equal amount of fine sand. The soil-sand mixture had available P of 10 mg kg^−1^ (sodium bicarbonate extractable), available K 57 mg kg^−1^ (ammonium acetate extractable), available Ca 798 mg kg^−1^ (ammonium chloride extractable), available Mg 30 mg kg^−1^ (ammonium acetate extractable), available Mn 5 mg kg^−1^ (magnesium nitrate extractable), available Cu 17 mg kg^−1^ (EDTA extractable), available Zn 15 mg kg^−1^ (EDTA extractable), and available B 45 mg kg^−1^ (EDTA extractable).

Nitrogen (N, in the form of NH_4_NO_3_) fertilizer at the rate of 100 kg N ha^−1^ was applied and mixed homogeneously into the entire volume of soil and sand mixture in all the tubes. Two plants were grown in each tube, and each genotype was replicated four times. The tubes were watered to soil water holding capacity initially and then watered to 80% of soil water holding capacity twice a week. The seeds germinated on June 12th 2012, and the plants were harvested on July 12th, 2012 [30 days after emergence (DAE)] at stem elongation growth stage. The soil columns were excavated into a water bath and then all the roots were washed out using a gentle stream of water with great care to minimize any potential damage to root hairs.

### Sampling, measurements, and analyses

Transparent plastic sheets with printed grid lines were placed on the surface of the Perspex tubes to measure *in situ* root intensity and depth. When not subjected to root recording, the tubes were covered with a black plastic sheet to prevent from light. Root intensity was calculated according to Thorup-Kristensen et al. (2009) and expressed as root intersections m^−1^ grid line. Root intensity was recorded five times during the experimental period. All the collected roots were scanned using a root scanner (Epson Perfection V700, CA, USA) and analyzed by the WinRHIZO image analysis system (Regents Instruments Inc., Quebec city, Canada) for root length and surface area. Randomly selected root segments in the root hair zone from each replicate were placed in a film of water in petri dishes. Root hair images were captured for all the genotypes on the main root axis and first order and second order roots, using a video camera fitted to a microscope (Leica Microsystems GmbH, Wetzlar, Germany) at 2.5 × magnification interfaced with a computer image grabber board. Root hairs were analyzed using ImageJ software. All root hairs were measured manually with the same procedure. Root hair length (RHL) was measured at more than 100 randomly selected root hairs for each replicate. Root hair density (RHD) was determined as the number of root hairs per mm root length. The total length of root hairs mm^−1^ root (TLRH) was calculated by multiplying RHL and RHD.

The DM of shoot, root and original seed material was determined after oven drying to constant weight at 70°C. After grinding, the samples were digested in a microwave oven with HNO_3_ and HCl in a 1:3 v:v mixture, then analyzed for P, K, Ca, Mg, S, Fe, Mn, Cu, Zn, and B using inductively coupled plasma-optical emission spectroscopy (ICP-OES; Optima 5300 DV, Perkin Elmer Inc., USA). Total N was analyzed using the Dumas dry combustion method in a system consisting of an ANCA-SL Elemental Analyser coupled to a 20–20 Mass Spectrometer (Sercon Instruments, Crewe, UK). Nutrient content was calculated as multiplication of concentration by dry matter with subtraction of seed reserve.

### Statistical analysis

The data were analyzed by one-way analysis of variance (ANOVA) using SAS GLM (SAS Institute, Inc., 2011) at a significance level of 5%. Duncan's multiple range test was applied to assess the differences between treatments at a significance level of 5%. Linear regression analyses were used to determine the relationships between the measured parameters.

## Results

### Root and root hair traits

**Figure 1:**
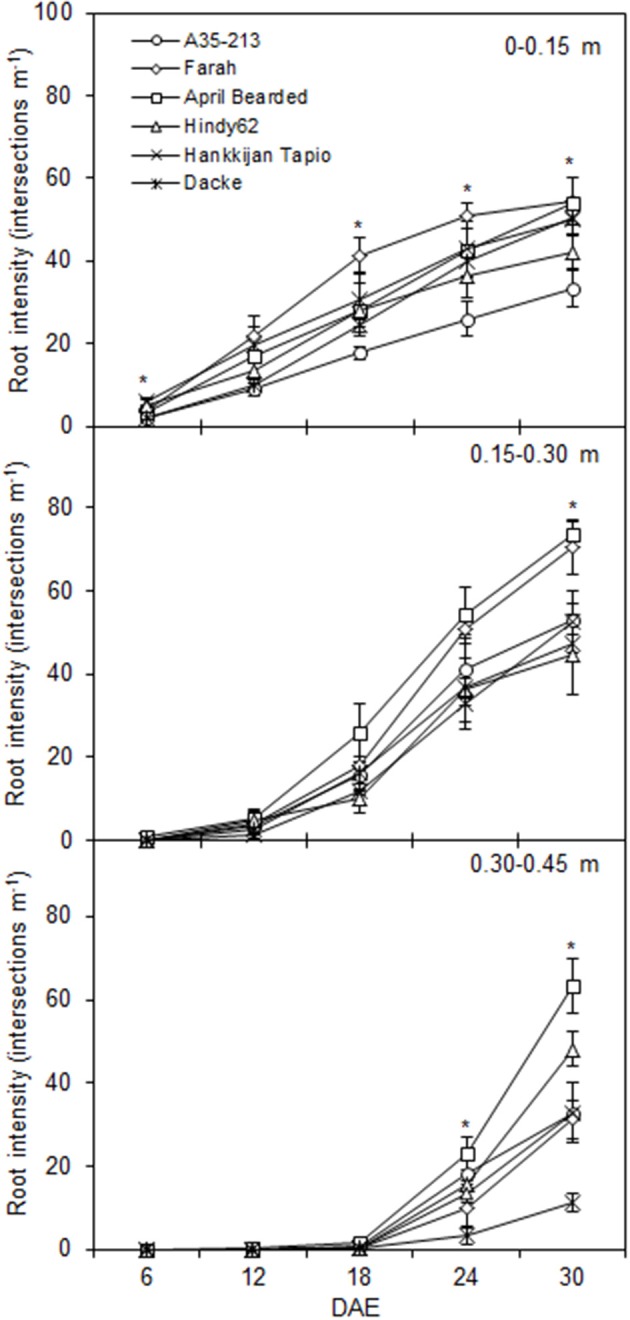
**Depth distribution of root intensity for spring wheat genotypes during the experimental period**. Values are means ± *SE* (*n* = 4). ^*^ indicates significant differences among genotypes at *P* < 0.05. DAE denotes days after emergence.

**Figure 2:**
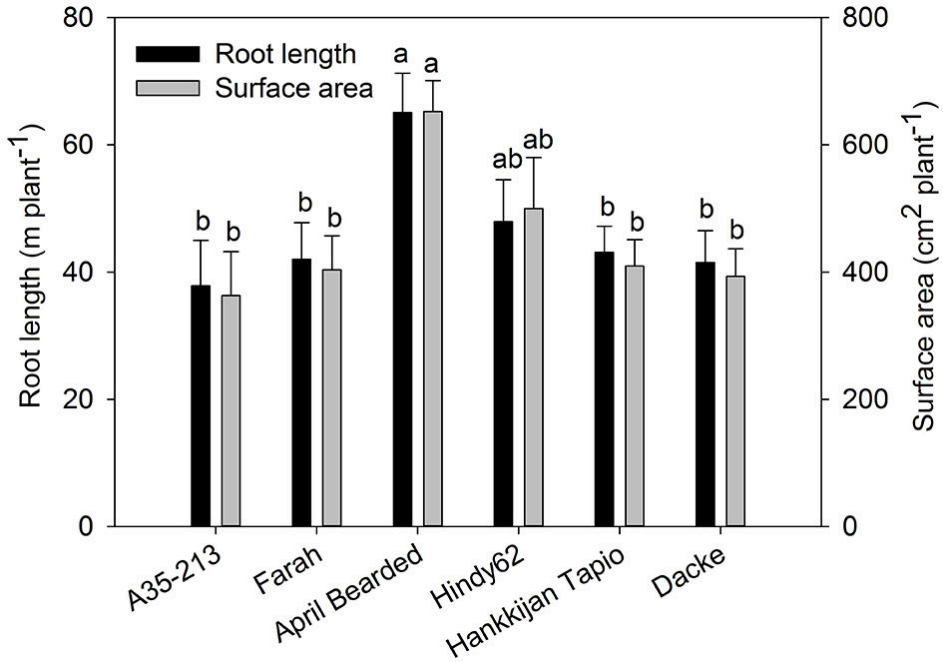
**Root length and root surface area of spring wheat genotypes**. Values are means ± *SE* (*n* = 4). Different letters indicate significant differences among genotypes according to Duncan's multiple range test at *P* < 0.05.

The root development over time differed markedly among genotypes (Figure 1), with April Bearded showing a more vigorous root growth. Total root length and root surface area differed significantly where April Bearded produced the largest root length and surface area, Hindy62 was intermediate, and the lowest were found for A35-213, Farah, Hankkijan Tapio and Dacke (Figure 2). The root length density was shown in Figure S2.

**Figure 3:**
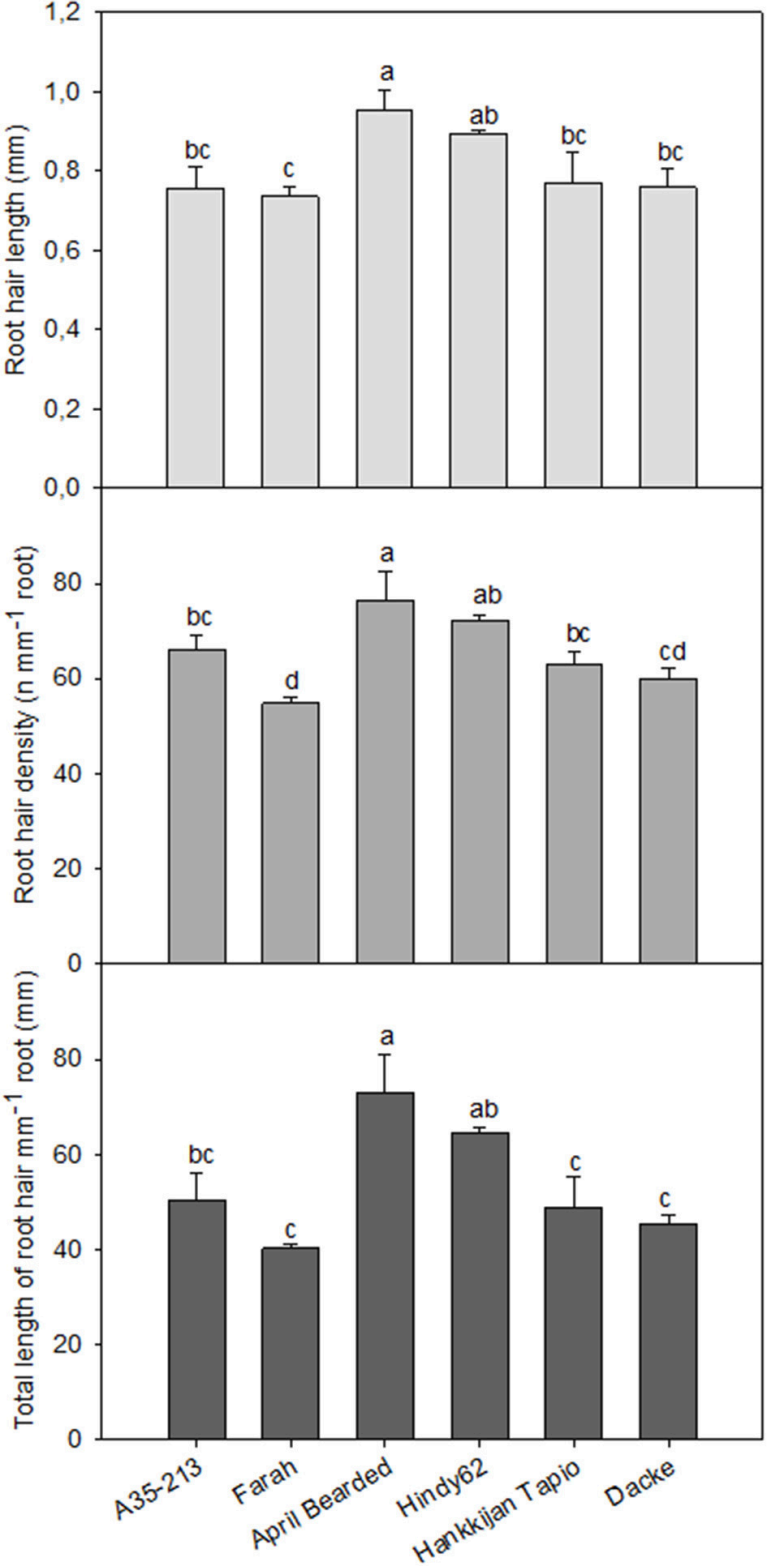
**Root hair length, root hair density, and total length of root hairs mm^**−1**^ root of spring wheat genotypes**. Values are means ± *SE* (*n* = 4). Different letters indicate significant differences among genotypes according to Duncan's multiple range test at *P* < 0.05.

Significant differences in RHL, RHD and TLRH among genotypes were also observed. The roots of April Bearded and Hindy62 were covered with the longest and densest root hairs, whereas Farah had the lowest RHL, RHD and TLRH (Figure 3).

### Plant growth and the ratio of root length and shoot dry matter

There were substantial and significant differences in shoot DM among genotypes (Table 2). Shoot DM was by far highest for April Bearded, intermediate for A35–213 and Hankkijan Tapio, and the lowest for Farah, Hindy62 and Dacke (Table 2). April Bearded, Hindy62, Farah, and Dacke had higher ratio of root length and shoot DM compared with A35–213 and Hankkijan Tapio.

**Table 2:** **Concentration and content of macronutrients, shoot dry matter (DM), and root length/shoot DM ratio of spring wheat genotypes**.

**Genotype**	**Macronutrient concentration (mg g^−1^)**						**Macronutrient content (mg plant^−1^)**						**Shoot DM (mg)**	**Root length/shoot DM (m g^−1^)**
	**N**	**P**	**K**	**Ca**	**Mg**	**S**	**N**	**P**	**K**	**Ca**	**Mg**	**S**		
A35-213	37.9bc	2.2b	39.8c	5.3abc	1.3bc	2.9a	26.3b	1.4b	28.3b	3.7ab	0.9b	2.0bc	710b	52c
Farah	40.3abc	2.6a	46.6a	5.6ab	1.3c	3.3a	24.4b	1.4b	29.2b	3.5b	0.7b	2.0bc	634bc	66ab
April Bearded	41.6ab	2.6a	44.8ab	4.6c	1.6a	3.2a	38.9a	2.3a	42.9a	4.4a	1.5a	3.0a	959a	67ab
Hindy62	42.9a	2.5a	42.3bc	6.0a	1.6ab	3.1a	28.0b	1.6b	28.7b	4.0ab	1.0b	2.1bc	684bc	70ab
Hankkijan Tapio	36.7c	2.2b	39.4c	4.9bc	1.2c	3.1a	26.7b	1.5b	29.6b	3.7ab	0.9b	2.3b	754b	58bc
Dacke	42.9a	2.6a	42.8bc	6.0a	1.7a	3.0a	22.7b	1.3b	23.3b	3.3b	0.9b	1.6c	546c	77a

*Different letters in the column indicate significant differences among genotypes according to Duncan's multiple range test at P < 0.05*.

### Concentrations and contents of macro- and micronutrients

**Figure 4:**
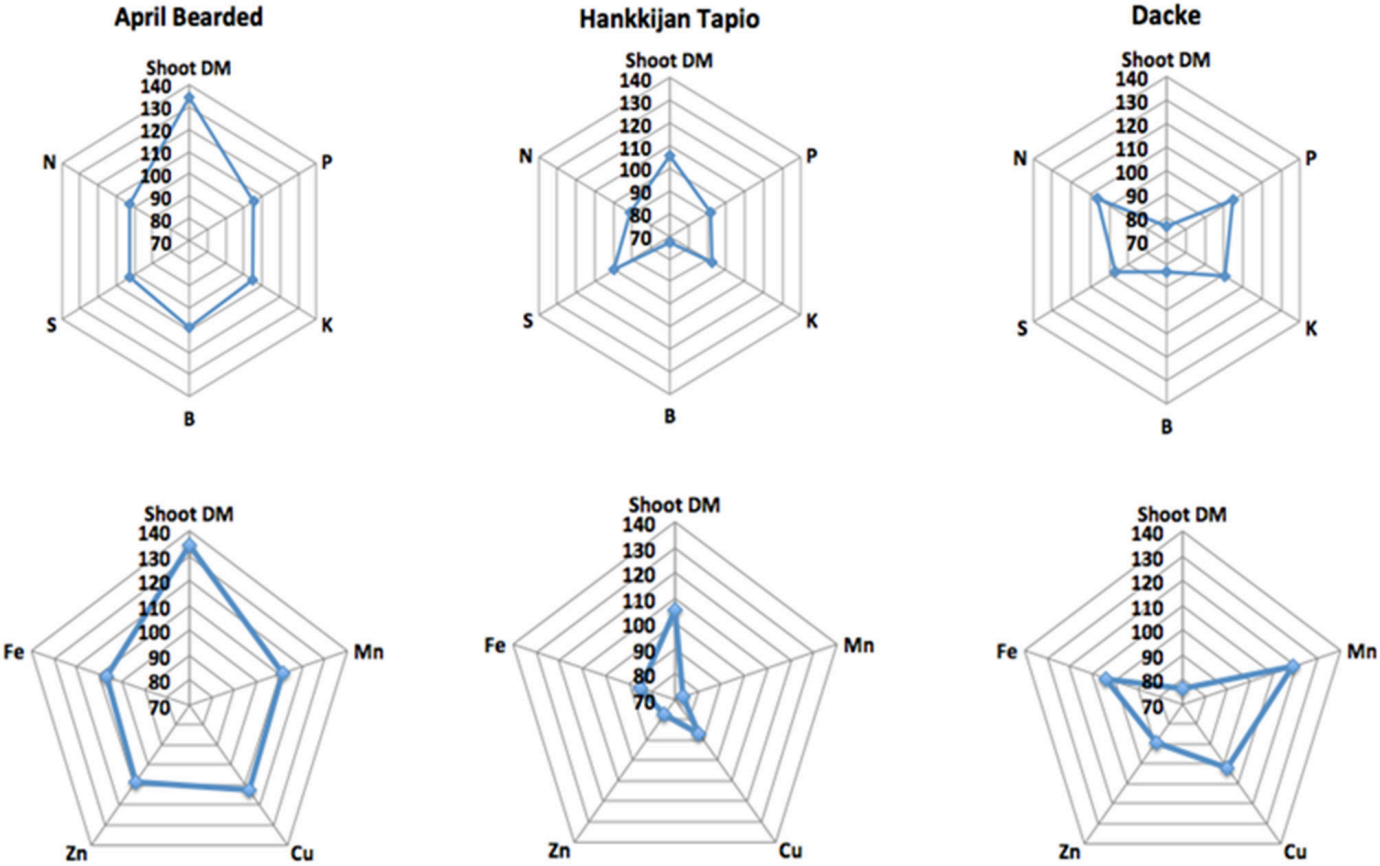
**Relative shoot dry matter (DM) and relative concentrations of nutrients in the three selected genotypes**.

The concentrations of macro- and micronutrients in the shoots showed remarkable differences among genotypes, the only exception being sulfur (S) with no significant difference. We analyzed the correlations between root length or root hair traits and nutrient concentrations, but no such correlations were found. However, concentrations of N, P, K, Ca, and Mg were higher in Farah, April Bearded, Hindy62 and Dacke compared to A35–213 and Hankkijan Tapio, except for lower Ca in April Bearded and lower Mg in Farah (Table 2). Generally, April Bearded and Hindy62 had the highest concentrations of Fe, Mn, Cu, Zn, and B (Table 3). This is illustrated in Figure 4, in which three selected genotypes were compared based on the averages of all six genotypes with respect to shoot DM and nutrient concentrations. Here, it was found that April Bearded combined the highest shoot production with higher than average concentrations of most nutrients, while Hankkijan Tapio had a better than average shoot production but rather low concentrations of most nutrients. Dacke, on the other hand, combined a poor shoot production with a better than average N, P, and K concentration, a low B and a high Mn concentration.

**Table 3:** **Concentration and content of micronutrients, shoot dry matter (DM), and root length/shoot DM ratio of spring wheat genotypes**.

**Genotype**	**Micronutrient concentration (μg g^−1^)**					**Micronutrient content (μg plant^−1^)**					**Shoot DM (mg)**	**Root length/shoot DM (m g^−1^)**
	**Fe**	**Mn**	**Cu**	**Zn**	**B**	**Fe**	**Mn**	**Cu**	**Zn**	**B**		
A35-213	81.0ab	16.4bc	5.8a	29.1bc	1.9a	56.5b	10.9b	4.0b	19.7b	1.4b	710b	52c
Farah	80.2ab	17.3abc	5.0b	30.9ab	1.5b	48.8b	10.1b	3.0c	18.4bc	1.0c	634bc	66ab
April Bearded	89.6a	20.4ab	6.3a	31.4ab	1.8a	84.1a	18.7a	5.9a	29.6a	1.7a	959a	67ab
Hindy62	94.2a	20.6ab	6.0a	34.2a	1.9a	61.6ab	12.4b	3.8bc	22.0b	1.3b	684bc	70ab
Hankkijan Tapio	71.1b	13.4c	4.9b	22.4d	1.2c	51.5b	9.4b	3.6bc	15.9bc	0.9c	754b	58bc
Dacke	87.1a	21.7a	5.7a	25.8cd	1.3c	46.0b	11.2b	3.0c	12.8c	0.7c	546c	77a

*Different letters in the column indicate significant differences among genotypes according to Duncan's multiple range test at P < 0.05*.

The content of macro- and micronutrients also differed significantly among genotypes. April Bearded generally absorbed the highest amount, A35–213, Farah, Hindy62, and Hankkijan Tapio were intermediate, and the lowest was Dacke (Tables 2, 3).

Significant positive linear relationships were found between: (1) root length and macro- and micronutrient content, and root surface area and macro- and micronutrient content, with the exception of B (Table 4); (2) RHL and RHD and most macro- and micronutrient content.

**Table 4:** **Linear regression coefficients between the means of measured plant macro- and micronutrient content and root variables**.

	**Root length**	**Surface area**	**Root hair length**	**Root hair density**
N	0.88^**^	0.87^**^	0.73^*^	0.69^*^
P	0.91^**^	0.88^**^	0.67^*^	0.58
K	0.82^*^	0.78^*^	0.54	0.47
Ca	0.72^*^	0.77^*^	0.80^*^	0.81^*^
Mg	0.88^**^	0.86^**^	0.78^*^	0.74^*^
S	0.75^*^	0.71^*^	0.51	0.47
Fe	0.85^**^	0.87^**^	0.81^*^	0.79^*^
Mn	0.89^**^	0.88^**^	0.75^*^	0.68^*^
Cu	0.76^*^	0.75^*^	0.68^*^	0.75^*^
Zn	0.73^*^	0.77^*^	0.71^*^	0.67^*^
B	0.47	0.52	0.57	0.70^*^

* and ** *indicate significance at P < 0.05 and P < 0.01, respectively (n = 6)*.

**Figure 5:**
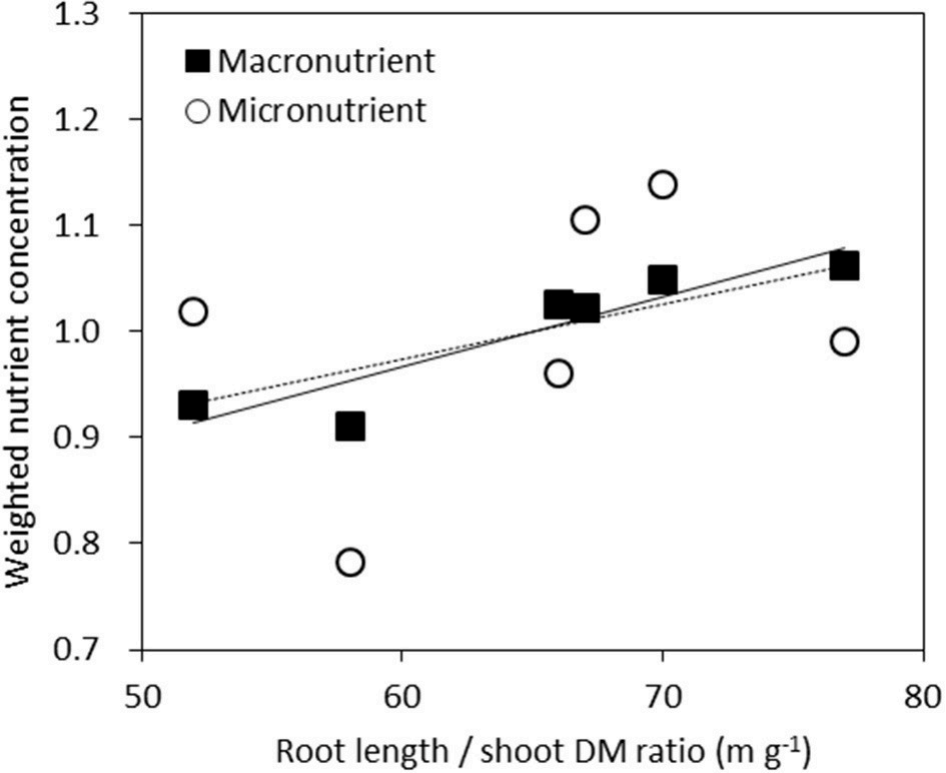
**Weighted average nutrient concentrations of genotypes plotted against their root length/shoot DM ratio**. For each nutrient the concentration of each genotype relative to the averaged concentration across the six genotypes was calculated. This value was then averaged across the six macronutrients N, P, K, Ca, Mg, and S (■, full line, *r*^2^ = 0.85, *P* < 0.01) or five micronutrients Fe, Mn, Cu, Zn, B (◦, dashed line *r*^2^ = 0.13, *P* = 0.48).

We examined the average nutrient concentrations of genotypes against their respective root length/shoot DM ratio. This may be seen as an indicator of the balance between root supply and shoot demand. For each nutrient the concentration of each genotype relative to the average concentration across the six genotypes was calculated. This value was then averaged across the six macronutrients of N, P, K, Ca, Mg, and S or five micronutrients of Fe, Mn, Cu, Zn, and B (Figure 5). The resulting correlation was highly significant (*r*^2^ = 0.85, *P* < 0.01) across macronutrients, but not significant across micronutrients (*r*^2^ = 0.13, *P* = 0.48).

## Discussion

Root traits influencing the acquisition of mineral elements include root length, root hair characteristics, and root distribution in soil layers, all of which increase the volume of soil explored by the root system and the surface area for uptake of mineral nutrients (White et al., 2013). Root hairs have been demonstrated to be important for P acquisition in a number of crop species, particularly in low-P environment (Foehse et al., 1991; Hoffmann and Jungk, 1995; Bates and Lynch, 1996, 2000; Gahoonia et al., 1997; Yan et al., 2004; Brown et al., 2012). However, the relative importance of root hairs to general nutrient acquisition during early growth stages and root system establishment is still unclear. In the current study, it was found that the accumulation of most macro- and micronutrient was significantly positively and linearly correlated with RHL and RHD (Table 4). In the current experiment, soil was sampled from a low yielding treatment which had not been fertilized for 9 years, and was further mixed with 50% sand, in order to allow isolation of roots. Thus, the fertility level in our experiment was low. Under low soil fertility, root hairs may function not only as an uptake system with a large surface area, but also facilitate the passage of less mobile nutrients through the soil (Jungk, 2001). In addition, water channels, as well as phosphate, potassium, calcium, and sulfate transporters are localized in root hairs, and it has been suggested that root hairs take part in the uptake of most macro- and micronutrients (Gilroy and Jones, 2000; Libault et al., 2010). It is noteworthy that RHL displayed a higher linear correlation with nutrient content than RHD for most nutrients, indicating more importance of RHL than RHD in facilitating nutrient acquisition. Consistent with this, Zygalakis et al. (2011) reported that increase in RHL presented a bigger advantage for nutrient uptake than increase in RHD. However, our results did not confirm that uptake of P was more dependent on root hair traits than N. To the best of our knowledge this finding has not been reported previously. This may be related to the fact that we studied the early growth phases where the ability of plants to spread the root system into not yet exploited parts of the soil volume is important for uptake of all nutrients before important root overlap and competition occur later. Consequently, the content of most macro- and micronutrients was significantly positively and linearly correlated with both root length and surface area (Table 4), and the linear correlation coefficients were higher than those between root hair traits and nutrient content, indicating that vigorous root growth was a better indicator than root hair traits of early nutrient uptake in spring wheat plants grown at low soil fertility.

Genotypes used in the current study showed significant differences in root distribution and depth during the experimental period, which may contribute to differences in nutrient and water uptake (Gregory, 2006). While nutrient concentrations are higher in the shallow soil horizon, selection of wheat genotypes on the basis of early and prolific root branching could improve the uptake of nutrients (Liao et al., 2006), and deeper root systems could increase water and N uptake from the subsoil (White and Kirkegaard, 2010). The genotypes with early vigorous root growth, long and dense root hairs, combined with early root distribution in topsoil and depth of rooting can be expected to capture a higher amount of macro- and micronutrients from low fertility soil, which synchronously supports higher biomass like April Bearded.

Higher macronutrient concentrations were related to a higher ratio of root length to shoot DM (Figure 5), indicating that vigorous roots enable plants to absorb higher amounts of macro- and micronutrients from soil, and thereby supply higher macro-nutrient concentration to the shoots.

However, while it is clear that a vigorous root growth can enable a more rapid depletion of nutrients in a low fertility soil, a number of other traits could be important for determining the genetic variability in the efficiency of nutrient uptake of the root system. Differences between cultivars could also be due to differences in the excretion of protons, organic acids and other exudates (Lynch, 2011; White et al., 2013).

Nutrient uptake is dependent on one hand on root nutrient uptake capacity by the root system but also on above-ground growth creating shoot nutrient demand. If nutrient uptake is stimulated by increased shoot demand without a concomitant increase in supply from roots, this will lead to reduced nutrient concentration in the plant due to dilution; this effect can be seen in Hankkijan Tapio and Dacke—where Hankkijan Tapio has higher shoot dry matter and total uptake of most nutrients but simultaneously lower concentrations (Figure 4), yet Dacke has higher concentrations but smaller shoot dry matter. This suggests that increased nutrient uptake by Hankkijan Tapio is caused mainly by non-root factors increasing growth and nutrient demand. If increased uptake of nutrients is facilitated by increased nutrient supply capacity in the root system, shoot growth may at least initially be accompanied by higher nutrient concentrations in plant tissue. To the best of our knowledge this finding has not been reported previously. This is evident with April Bearded, which had above average concentrations of most nutrients, even though it also had the strongest growth of all the genotypes. Our study also showed that there were significant positive genetic correlations between root and root hair traits and nutrient content (data not shown), indicating functional links between root traits and nutrient uptake, and showing that selection for improved root traits during early growth may be possibly be employed for breeding of nutrient efficient spring wheat genotypes for low-input and organic agriculture.

## Conclusions

Our results show clearly that there was a significant genetic variability in root and root hair traits under low soil nutrient availability. A high root length to shoot dry matter ratio favored high concentration of macronutrients in the shoots. Vigorous root growth and long and dense root hairs are important traits in ensuring efficient acquisition of both macro- and micronutrients in the early establishment of spring wheat in nutrient-limited soil and low nutrient input cropping systems.

## Author contributions

JM, KT, and LS formulated the research questions. YW, JM, KT, and LS designed the experiment. YW performed the experiments, analyzed the data and drafted the manuscript. JM, KT, and LS participated in the data analysis, assisted with the revisions to the manuscript and coordination of the study.

### Conflict of interest statement

The authors declare that the research was conducted in the absence of any commercial or financial relationships that could be construed as a potential conflict of interest. The reviewer JK and handling Editor declared their shared affiliation, and the handling Editor states that the process nevertheless met the standards of a fair and objective review.
