# Supplementary material for: Vigorous Root Growth Is a Better Indicator of Early Nutrient Uptake than Root Hair Traits in Spring Wheat Grown under Low Fertility
Source: Front Plant Sci. 2016 Jun 16;7:865. doi: 10.3389/fpls.2016.00865 (PMC4910668; doi:10.3389/fpls.2016.00865)
Supplement: Supplementary file 1 [file Table1.DOCX]

**Table S1** The root length, root hair length and origin of 19 spring wheat genotypes tested in the preliminary experiment of this study.

| Genotype | Root length (m) | Root hair length (mm) | Origin |
| --- | --- | --- | --- |
| A35-213 | 45 | 0.58 | Norway |
| HANKKIJAN TAPIO | 48 | 0.55 | Finland |
| BASTIAN | 54 | 0.81 | Norway |
| HJA 21048 | 57 | 0.64 | Finland |
| DRABANT | 57 | 0.74 | Sweden |
| VINJETT | 57 | 0.54 | Sweden |
| DRAGON | 57 | 0.51 | Sweden |
| HINDY62 | 57 | 0.81 | Egypt |
| AMARETTO | 60 | 0.57 | Germany |
| AURORE | 61 | 0.50 | Australia |
| HALLE 13471 | 61 | 0.55 | Denmark |
| DACKE | 63 | 0.45 | Sweden |
| HALLAND | 64 | 0.56 | Sweden |
| TRISO | 65 | 0.71 | Germany |
| BØRSUM | 67 | 0.54 | Norway |
| DALARNA | 67 | 0.55 | Sweden |
| FARAH | 70 | 0.77 | Afghanistan |
| ASOSAN X EDERATION BC3 | 73 | 0.58 | Denmark |
| APRIL BEARDED | 79 | 0.65 | United Kingdom |

The identities of spring wheat genotypes are available at NordGen ([www.nordgen.org](http://www.nordgen.org))
