# Supplementary material for: Vigorous Root Growth Is a Better Indicator of Early Nutrient Uptake than Root Hair Traits in Spring Wheat Grown under Low Fertility
Source: Front Plant Sci. 2016 Jun 16;7:865. doi: 10.3389/fpls.2016.00865 (PMC4910668; doi:10.3389/fpls.2016.00865)
Supplement: Supplementary file 2 [file Table2.DOCX]

**Table S2** Root dry matter (DM) of spring wheat genotypes. Different letters in the column indicate significant differences between genotypes according to Duncan’s multiple range test at *P* < 0.05

| **Genotype** | **Root DM (mg)** |
| --- | --- |
| A35-213 | 190b |
| Farah | 195b |
| April Bearded | 298a |
| Hindy62 | 251ab |
| Hankkijan Tapio | 208b |
| Dacke | 206b |
