# Supplementary material for: Vigorous Root Growth Is a Better Indicator of Early Nutrient Uptake than Root Hair Traits in Spring Wheat Grown under Low Fertility
Source: Front Plant Sci. 2016 Jun 16;7:865. doi: 10.3389/fpls.2016.00865 (PMC4910668; doi:10.3389/fpls.2016.00865)

Figure S1 Examples of analyzed images for measurement of root hair length and density of spring wheat genotypes.

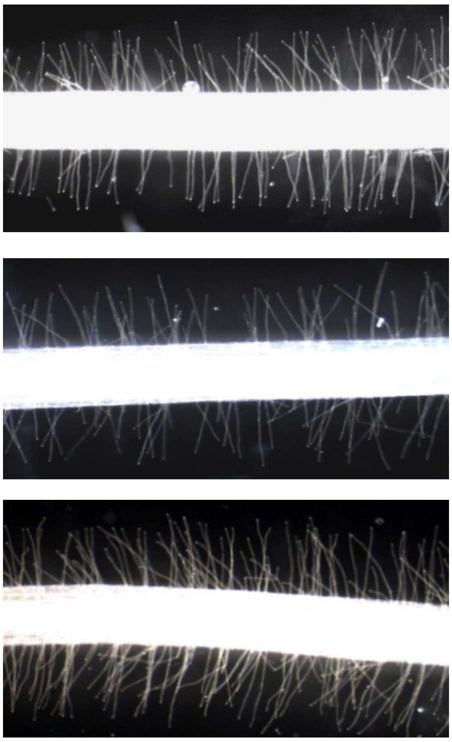

Supplement: Supplementary file 3 [file Image1.PDF]
