# Supplementary material for: Vigorous Root Growth Is a Better Indicator of Early Nutrient Uptake than Root Hair Traits in Spring Wheat Grown under Low Fertility
Source: Front Plant Sci. 2016 Jun 16;7:865. doi: 10.3389/fpls.2016.00865 (PMC4910668; doi:10.3389/fpls.2016.00865)

Figure S2 Root length density of spring wheat genotypes in the pots (two plants per pot). Different letters above the column indicate significant differences between genotypes according to Duncan's multiple range test at $P < 0.05$

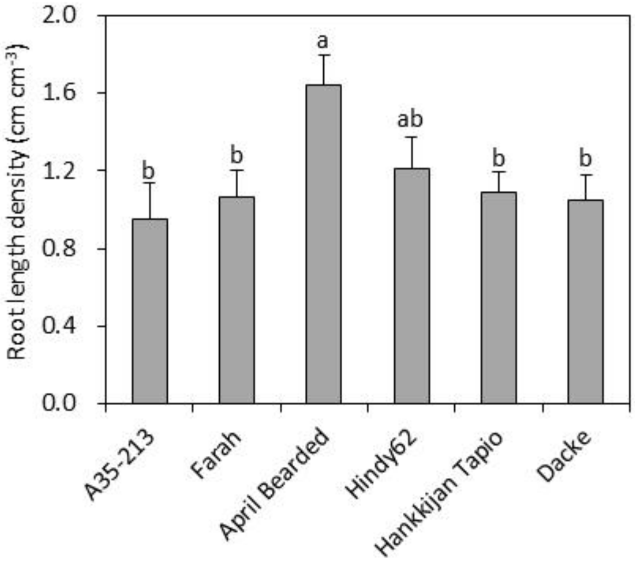

Supplement: Supplementary file 4 [file Image2.PDF]
